# Attributable Risk and Consequences of Bone Mineral Density Deficits in Childhood Cancer Survivors

**DOI:** 10.1001/jamanetworkopen.2024.54069

**Published:** 2025-01-10

**Authors:** Chelsea G. Goodenough, Jessica L. Baedke, Angela M. Delaney, Carmen L. Wilson, Tara M. Brinkman, Cindy Im, Megan E. Ware, Hiroto Inaba, Karen L. Clark, Gregory T. Armstrong, Daniel A. Mulrooney, Ching-Hon Pui, Daniel M. Green, Thomas E. Merchant, Deo Kumar Srivastava, Yutaka Yasui, Melissa M. Hudson, Leslie L. Robison, Sue C. Kaste, Kirsten K. Ness, Wassim Chemaitilly

**Affiliations:** 1 Department of Epidemiology and Cancer Control, St Jude Children’s Research Hospital, Memphis, Tennessee; 2 Department of Psychology and Biobehavioral Sciences, St Jude Children’s Research Hospital, Memphis, Tennessee; 3 Endocrinology Department, St Jude Children’s Research Hospital, Memphis, Tennessee; 4 Oncology Department, St Jude Children’s Research Hospital, Memphis, Tennessee; 5 Department of Pediatrics, University of Minnesota, Minneapolis; 6 Center for Advanced Practice, St Jude Children’s Research Hospital, Memphis, Tennessee; 7 Department of Radiation Oncology, St Jude Children’s Research Hospital, Memphis, Tennessee; 8 Biostatistics Department, St Jude Children’s Research Hospital, Memphis, Tennessee; 9 Diagnostic Imaging Department, St Jude Children’s Research Hospital, Memphis, Tennessee; 10 Division of Endocrinology, Department of Pediatrics, University of Pittsburgh School of Medicine, Pittsburgh, Pennsylvania

## Abstract

**Importance:**

Data characterizing the severity and changing prevalence of bone mineral density (BMD) deficits and associated nonfracture consequences among childhood cancer survivors decades after treatment are lacking.

**Objective:**

To evaluate risk for moderate and severe BMD deficits in survivors and to identify long-term consequences of BMD deficits.

**Design, Setting, and Participants:**

This cohort study used cross-sectional and longitudinal data from the St Jude Lifetime (SJLIFE) cohort, a retrospectively constructed cohort with prospective follow-up. Participants in SJLIFE are adult survivors of childhood cancer who were diagnosed between 1962 and 2012 and survived 5 years or more from diagnosis. Data were collected from November 2007 to June 2020 and analyzed from January 2021 to November 2023.

**Exposures:**

Childhood cancer therapy exposures, clinically ascertained comorbid conditions, substance use, and sedentary lifestyle.

**Main Outcomes and Measures:**

BMD was evaluated using lumbar quantitative computed tomography and classified by age- and sex-specific *z* scores with moderate (≤−1 SD) or severe (≤−2 SD) deficits. Multivariable logistic regression estimated odds ratios (ORs), attributable fractions (AFs), and associations between BMD deficits and long-term sequelae (social, functional, and quality of life [QOL]).

**Results:**

Among 3919 five-year survivors (median [range] age, 31.7 [18.0-69.9] years; 2063 [52.6%] male; 105 [2.7%] Hispanic, 607 [15.5%] non-Hispanic Black, and 3153 [80.4%] non-Hispanic White), prevalence of moderate or severe BMD deficits were 21.7% (95% CI, 20.4%-23.0%) and 6.9% (95% CI, 6.1%-7.7%), respectively. Treatment exposures (including age at diagnosis), comorbid conditions, and smoking and sedentary behavior explained 18.5%, 10.2%, and 7.0% of moderate and 55.4%, 51.1%, and 9.9% of severe deficits. Severe deficits were associated with 30 Gy or greater cranial radiotherapy (CRT) (OR, 5.22; 95% CI, 3.74-7.30; AF, 33.0%), testicular or pelvic radiation (OR, 1.70, 95% CI, 1.19-2.44; AF, 11.5%), hypogonadism (OR, 3.27, 95% CI, 2.35-4.55; AF, 25.1%), growth hormone deficiency (OR, 5.28, 95% CI, 3.68-7.56; AF, 26.0%), smoking (OR, 1.71, 95% CI, 1.21-2.43; AF, 6.7%), and sedentary behavior (OR, 2.06, 95% CI, 1.15-3.69; AF, 6.2%). CRT exposure increased risk for declining BMD (OR, 2.94, 95% CI, 1.46-5.91; AF, 8.8%). Survivors with deficits were less likely to live alone and to be employed and more likely to require personal care assistance and to report depressive symptoms and poor QOL.

**Conclusions and Relevance:**

While treatment exposures were associated with long-term BMD deficits, modifiable risk factors, including smoking, sedentary behavior, hypogonadism, and growth hormone deficiency, suggest feasible targets for intervention.

## Key Points

**Question:**

What are moderate and severe bone mineral density deficits attributable to in survivors of childhood cancer, and are these deficits associated with long-term consequences decades after treatment has ended?

**Findings:**

In this study of 3919 survivors of childhood cancer, treatment exposures, comorbid conditions, and smoking and sedentary behavior explained 18.5%, 10.2%, and 7.0% of moderate and 55.4%, 51.1%, and 9.9% of severe BMD deficit cases, respectively. Survivors with deficits were less likely to live alone or to be employed and more likely to require assistance with personal care and to report depressive symptoms and poor quality of life.

**Meaning:**

Findings of this study highlight the social, functional, and quality-of-life challenges survivors with BMD deficits persistently face; most deficits observed were directly attributable to treatment exposures, comorbidities, smoking, and sedentary behavior.

## Teaser

This cohort study examines the risk of moderate and severe bone mineral density deficits among survivors of childhood cancers and evaluates the social, functional, and quality-of-life outcomes of these deficits.

## Introduction

Although 5-year survival rates for childhood cancer exceed 85%,^1^ survivors are at risk for early-onset chronic health conditions, including altered skeletal health.^2,3,4^ Bone mineral density (BMD), a measure of structural integrity, captures the impact of lifetime exposures and habits on the skeleton. Peak bone mass acquisition occurs during childhood and adolescence, a time when children with cancer are exposed to aggressive therapies capable of altering bone metabolism and hormone production. Thirty percent of survivors have clinically ascertained low BMD within 5 years of diagnosis.^5^

Survivors who present with poor bone quality at diagnosis or early after cancer treatment may be at risk for additional bone loss related to health behaviors,^5,6^ including diet,^7,8,9^ weight-bearing activities,^10,11^ smoking, and alcohol consumption.^12,13^ Unfortunately, survivors have suboptimal nutrient intake^14,15^ and exercise habits^16^ compared with peers, which may alter the trajectory of BMD decline with age. Additionally, chronic health conditions may contribute to declining skeletal health during survivorship.

The severity and trajectory of BMD deficits are well-described in survivor cohorts in the first 5 to 10 years after cancer therapy.^17,18,19^ Although long-term consequences of prolonged exposure to poor bone quality may be substantial,^20^ no data exist to characterize the changing prevalence and severity of objectively measured BMD deficits in a large, diverse population of adults who survived childhood cancer.

The purpose of this study was to evaluate BMD deficit as a distinct health outcome in the St Jude Lifetime (SJLIFE) cohort during long-term survivorship. The aims were to (1) determine the prevalence, severity, and longitudinal trajectory of objectively measured BMD deficits; (2) quantify attributable risk of demographic characteristics, treatment, and lifestyle to BMD deficits and change in BMD deficits; and (3) evaluate the association of BMD deficits with functional independence and quality of life (QOL) in childhood cancer survivors.

## Methods

### Participants

Participants were recruited from the St Jude Children’s Research Hospital institutional review board–approved SJLIFE cohort, a retrospectively constructed cohort with prospective follow-up of health outcomes among individuals surviving pediatric cancer (NCT00760656).^21,22^ Participants provided written informed consent prior to participating in any study activities. For this analysis, eligible participants included survivors at least 5 years from childhood cancer diagnosis and aged 18 years or older at recruitment. Female participants were not eligible if pregnant or prior to 6 months post partum. Of 5182 potentially eligible participants, 15 (0.29%) were excluded due to previous enrollment in an intervention study to improve bone density.^23,24^

### Outcomes of Interest

#### BMD Deficit

BMD was evaluated using quantitative computed tomography (QCT; GE VCT LightSpeed 64-detector CT scanner [GE Healthcare]). Data were analyzed with calibration phantoms and software (Mindways, Inc).^25,26^ Average volumetric trabecular BMD calculations for lumbar vertebrae L1 and L2 were generated and reported as age- and sex-specific *z* scores.^27^ BMD status was classified as normal (>−1 SD), moderate deficit (≤−1 SD to >−2 SD) or severe deficit (≤−2 SD). SJLIFE participants receive BMD evaluations at baseline (≥5 years after diagnosis) and at subsequent SJLIFE assessments every 3 to 5 years. In a subsample of study participants with multiple SJLIFE assessments, change in BMD status (maintain, improve, decline) was defined as change in BMD status from baseline to most recent re-evaluation. Maintain was defined as no change; improve was defined as a change from moderate or severe to normal. Decline was defined as a change from normal to moderate or severe or a change from moderate to severe.

#### Functional and Social Independence

Functional and social independence were self-reported. These measures included educational attainment, employment, need for personal care assistance, and independent living.

#### Depressive Symptoms

Participants completed the Brief Symptom Inventory 18 (BSI-18).^28^ Responses were converted to T scores; scores of 63 or greater were classified as reporting depressive symptoms.

#### Health-Related QOL

QOL was assessed using the Medical Outcomes Survey 36-Item Short Form,^29^ providing 8 subscales. Age- and sex-specific normative data were used to calculate T scores. Scores of 40 or less were classified as poor QOL.

### Exposures

#### Diagnosis and Treatment

Diagnosis, treatment history, and therapeutic exposures were abstracted from medical records. Radiotherapy exposure to the brain (cranial radiotherapy [CRT]), testes, pelvis, abdomen, and spine were classified as yes if any part of the organ or region was in the treatment field. Total body irradiation was classified as yes if the whole body was treated with radiation for hematopoietic stem cell transplantation. Cumulative radiation dose to the hypothalamus and pituitary region of the brain was estimated by summing all treatments.^30^ Chemotherapy exposures were characterized by summing agents to determine cumulative doses for alkylating agents (cyclophosphamide equivalent dose),^31^ methotrexate, and glucocorticoids. Treatment with a calcineurin inhibitor was classified as yes among those treated with cyclosporine.

#### Demographic Characteristics, Substance Use, Activity, and Diet

Demographic variables included sex, race, ethnicity, and age. Because bone density varies by race and ethnicity in the general population, self-reported race and ethnicity were categorized for analysis as Hispanic, non-Hispanic Black, non-Hispanic White, and additional groups (including American Indian or Alaska Native, Asian, and Pacific Islander islanders as well as those whose race and ethnicity were not reported). Substance use included tobacco and/or marijuana use (current, previous, and never), heavy alcohol consumption (>5 drinks/d among male participants; >4 drinks/d among female participants),^32^ Activity metrics included physically active (≥150 min/wk of moderate or vigorous physical activity, as reported on the National Health and Nutrition Examination Survey Physical Activity Questionnaire),^33^ sedentary behavior (reporting rare or no physical activity), and resistance training (reporting activities to increase muscle strength). Diet, including daily caloric intake (in kilocalories), dietary calcium (in milligrams), magnesium (in milligrams), vitamin D (in milligrams), vitamin A (in micrograms), and caffeine (in milligrams) was assessed with the 1998 Block Food Frequency Questionnaire.^34^

#### Comorbidities

Adult body mass index (BMI [calculated as weight in kilograms divided by height in meters squared]) was adjusted for amputation status. Hypogonadism and growth hormone deficiency (GHD) were defined and graded per a modified version of the National Cancer Institute’s Common Terminology Criteria for Adverse Events (CTCAE) version 5,^3^ using information from medical records and clinical laboratory examinations and classified as present if grade 2 or greater. Vitamin D status was determined from serum 25-hydroxy-vitamin D (25[OH]D) in nanograms per milliliter.^35^

### Statistical Analysis

Descriptive statistics characterized demographic and treatment variables. Comparisons between participants and nonparticipants were made using the χ^2^ test or 2-sample *t* test as appropriate.

In cross-sectional analysis, the prevalence of moderate and severe BMD deficits and their associations with demographic characteristics, lifestyle, cancer treatment exposures, and comorbid conditions were assessed by using BMD status at the most recent evaluation. Bivariate logistic regression was used to explore crude associations between BMD deficits and risk factors. Factors having *P* values of .05 or less or identified a priori as clinically important were retained in final multivariable models. Because treatment exposures and comorbid conditions are highly correlated, separate models were constructed to estimate odds ratio (ORs) and 95% CIs for moderate and severe deficit risk. Model 1 included treatment exposures, substance use, activity, and diet, adjusted for demographic characteristics. Model 2 included comorbid conditions, substance use, activity, and diet, adjusted for demographic characteristics. Attributable fractions (AFs) for covariates significantly associated with increased risk for BMD deficits in the treatment or comorbid condition multivariable models were calculated.^36^

Logistic regression adjusting for sex, race and ethnicity, diagnosis age, assessment age, and diagnosis group were used to evaluate associations between moderate and severe deficits and long-term outcomes, including functional and social independence, depression, and poor QOL. Associations between baseline demographic characteristics, substance use, activity, diet, cancer treatment exposures, comorbid conditions, and change in BMD status were evaluated in separate multivariable logistic models, adjusted for mean time between evaluations. Factors associated with BMD status change (*P* < .05) in bivariate models or identified a priori as clinically important were retained in final models. For participants with normal BMD at baseline, the odds of declining to either moderate or severe BMD deficit was compared with maintaining normal BMD. For participants with moderate deficits at baseline, a multinomial model calculated the odds of declining to severe BMD status or improving to normal BMD status compared with maintaining moderate BMD. For participants with severe deficits at baseline, a multivariable logistic regression model was used to calculate the odds of improving to normal BMD status compared with maintaining any BMD deficit. Treatment and comorbid health conditions were evaluated in separate models when evaluating BMD status changes. AFs were calculated in the treatment or comorbid condition multivariable models for BMD decline among individuals with normal BMD at baseline.^36^ Analyses were performed using SAS statistical software version 9.4 (SAS Institute).

## Results

### Participants

The cross-sectional analysis included 3919 survivors (median [range] age, 31.7 [18.0-69.9], 2063 [52.6%] male; 105 [2.7%] Hispanic, 607 [15.5%] non-Hispanic Black, and 3153 [80.4%] non-Hispanic White) (eFigure 1 in Supplement 1). Participants and nonparticipants did not differ by race and ethnicity or primary diagnosis. Participants were more likely to be male and treated with glucocorticoids. Evaluation of BMD status change between baseline and most-recent evaluation included 1607 survivors with more than 1 BMD assessment (Table 1).

**Table 1.** Characteristics of Participant vs Nonparticipant Survivors

Characteristic	Individuals, No. (%)			*P* value	
	Nonparticipants (n = 1248)	Participants			
		Cross-sectional (n = 3919)	Longitudinal (n = 1607)	Cross-sectional vs nonparticipants	Cross-sectional vs longitudinal
Sex					
Male	697 (55.9)	2063 (52.6)	816 (50.8)	.05	.22
Female	551 (44.2)	1856 (47.4)	791 (49.2)		
Race and ethnicity					
Hispanic	31 (2.5)	105 (2.7)	47 (2.9)	.87	.30
Non-Hispanic Black	184 (14.7)	607 (15.5)	218 (16.6)		
Non-Hispanic White	1014 (81.3)	3153 (80.5)	1322 (82.3)		
Additional groups^a^	19 (1.5)	54 (1.4)	20 (1.2)		
Age at diagnosis, y					
0-4	460 (36.9)	1366 (34.9)	584 (36.3)	.04	<.001
5-9	313 (25.1)	903 (23.0)	394 (24.5)		
10-14	251 (20.1)	951 (24.3)	381 (23.7)		
15-19	212 (17.0)	662 (16.9)	248 (15.4)		
≥20	12 (1.0)	37 (0.9)	0		
Primary cancer diagnosis					
Acute lymphoblastic leukemia	343 (27.5)	1185 (30.2)	626 (39.0)	.11	<.001
Acute myeloid leukemia	51 (4.1)	151 (3.9)	56 (3.5)		
Bone cancer	36 (2.9)	154 (3.9)	80 (5.0)		
Central nervous system	141 (11.3)	528 (13.5)	144 (9.0)		
Germ cell tumor	35 (2.8)	82 (2.1)	37 (2.3)		
Hodgkin lymphoma	161 (12.9)	449 (11.5)	153 (9.5)		
Neuroblastoma	61 (4.9)	161 (4.1)	57 (3.6)		
Non-Hodgkin lymphoma	103 (8.3)	275 (7.0)	131 (8.2)		
Retinoblastoma	42 (3.4)	121 (3.1)	28 (1.7)		
Soft tissue sarcoma	122 (9.8)	362 (9.2)	130 (8.1)		
Wilms tumor	78 (6.3)	227 (5.8)	101 (6.3)		
Other	75 (6.0)	224 (5.7)	64 (4.0)		
Age at most recent SJLIFE evaluation, y					
18-25	NA	1053 (26.9)	119 (7.4)	NA	<.001
26-35		1462 (37.3)	651 (40.5)		
36-45		949 (24.2)	557 (34.7)		
46-55		386 (9.9)	280 (17.4)		
>55		69 (1.8)	0		
Cranial radiotherapy					
None	437 (39.7)	1794 (46.3)	1061 (66.4)	<.001	<.001
>0 and <30 Gy	543 (49.3)	1644 (42.4)	402 (25.2)		
≥30 Gy	122 (11.1)	441 (11.4)	134 (8.4)		
Testicular or pelvic irradiation					
None	805 (74.5)	3144 (80.2)	1320 (82.1)	<.001	.11
Yes	276 (25.5)	775 (19.8)	287 (17.9)		
Total body irradiation					
No	1074 (96.8)	3796 (96.9)	1566 (97.5)	.94	.28
Yes	36 (3.2)	123 (3.1)	41 (2.5)		
Abdominal radiation					
No	818 (73.6)	3089 (78.8)	1311 (81.6)	<.001	.02
Yes	294 (26.4)	830 (21.2)	296 (18.4)		
Spinal radiation					
No	990 (89.1)	3544 (90.4)	1473 (91.7)	.21	.17
Yes	121 (10.9)	375 (9.6)	134 (8.3)		
Hypothalamic or pituitary radiotherapy					
No	763 (69.6)	2747 (71.2)	1079 (67.1)	.32	.004
Yes	334 (30.5)	1114 (28.9)	528 (33.1)		
Alkylating agents, mg/m^2^					
0	391 (48.8)	1860 (47.6)	717 (44.4)	.53	.09
>0 to <4000	108 (13.5)	499 (12.8)	198 (12.4)		
≥4000 to <8000	132 (16.5)	620 (15.9)	264 (16.5)		
≥8000	171 (21.3)	926 (23.7)	428 (26.7)		
Methotrexate					
None	462 (37.0)	2020 (51.5)	658 (41.0)	<.001	<.001
Yes	715 (57.3)	1394 (35.6)	716 (44.5)		
High dose (≥20 000 mg/m^2^)	71 (5.7)	505 (12.9)	233 (14.5)		
Glucocorticoid dose, mg/m^2^					
0	485 (60.7)	2130 (54.9)	736 (45.8)	.001	<.001
>0 to <3240	162 (20.3)	777 (20.0)	398 (24.8)		
≥3240	152 (19.0)	970 (25.0)	473 (29.4)		
Intrathecal chemotherapy					
None	699 (60.6)	2409 (61.5)	851 (53.0)	.61	<.001
Yes	455 (39.4)	1510 (38.5)	756 (47.0)		
Hematopoietic stem cell transplant					
No	1157 (92.7)	3607 (92.0)	1526 (95.0)	.48	<.001
Yes	91 (7.3)	312 (8.0)	81 (5.0)		

Abbreviations: NA, not applicable; SJLIFE, St Jude Lifetime Cohort.

^a^ The additional race and ethnicity group includes American Indian or Alaska Native, Asian, and Pacific Islander islanders as well as those whose race and ethnicity were not reported.

### Prevalence of BMD Deficits

Of 3919 participants included in the cross-sectional analyses, 849 (21.7%) had moderate and 271 (6.9%) had severe BMD deficits at their most recent BMD evaluation (median [IQR] survival 23.0 [16.4-31.4] years) (eFigures 2 and 3 in Supplement 1). Moderate (559 of 2063 [27.1%] vs 290 of 1856 [15.6%]) and severe (182 [8.8%] vs 89 [4.8%]) BMD deficits were higher in male than in female participants (*P* < .001).

Of 1607 survivors who had multiple BMD evaluations (median [IQR] survival, 22.5 [17.0-28.7] years), 316 (20.9%) had moderate and 91 (5.9%) had severe BMD deficits at baseline BMD evaluation (Table 2). Of the 1200 survivors with normal BMD at baseline, 98 (8.0%) developed moderate or severe BMD deficits. Of the 316 survivors with a moderate BMD deficit at baseline evaluation, 206 (65.0%) maintained a moderate deficit, 36 (11.0%) declined to a severe deficit, while 74 (23.0%) improved to normal BMD status. Of 91 survivors who had severe BMD deficits at baseline, only 1 (1.0%) improved to normal BMD status. Median (IQR) time between BMD evaluations was 5.4 (4.8-6.2) years.

**Table 2.** Participants Whose BMD Status Changed Between First and Most Recent BMD

Baseline BMD status	Total, No. (column %) (n = 1607)	BMD trajectory, No. (row %)^a^		
		Maintain	Improve	Decline
Normal (>−1 SD)	1200 (74.7)	1102 (92.0)	NA	98 (8.0)
Moderate (<−1 SD to >−2 SD)	316 (19.7)	206 (65.0)	74 (23.0)	36 (11.0)
Severe (≤−2 SD)	91 (5.7)	90 (99.0)	1 (1.0)	NA

Abbreviations: BMD, bone mineral density; NA, not applicable.

^a^ The definitions of maintain, improve, and decline appear in the Methods section.

### Treatment Exposures, Substance Use, Activity, Diet, and BMD Deficits

Compared with those with normal BMD, survivors with moderate deficits were more likely to be current smokers, aged 5 to 9 years at diagnosis, or exposed to 30 Gy or greater CRT or to 3240 mg/m^2^ or greater of glucocorticoids. These variables jointly had an AF of 24.6% for the 849 survivors with moderate BMD deficit in this model focused on treatment exposures, with 18.5% for age at diagnosis and exposure to 30 Gy or greater CRT or 3240 mg/m^2^ or greater of glucocorticoids (Table 3).

**Table 3.** Treatment Exposures, Lifestyle, and Moderate and Severe BMD Deficit Risk at Most Recent BMD Evaluation^a^

Factor	BMD deficit								
	Moderate (n = 849)				Severe (n = 271)				
	No. (%)	OR (95% CI)	*P* value	AF, %	No. (%)	OR (95% CI)	*P* value	AF, %
Race/ethnicity								
Non-Hispanic White	768 (90.46)	1 [Reference]	NA	NA	247 (91.14)	1 [Reference]	NA	NA
Hispanic	25 (2.94)	0.79 (0.47-1.32)	.37	NC	13 (4.80)	1.20 (0.60-2.39)	.61	NC
Non-Hispanic Black	43 (5.06)	0.20 (0.14-0.29)	<.001	NC	8 (2.95)	0.10 (0.05-0.22)	<.001	NC
Additional groups	13 (1.53)	0.67 (0.32-1.41)	.29	NC	3 (1.11)	0.53 (0.15-1.88)	.32	NC
Age at diagnosis, y								
0-4	267 (31.45)	1 [Reference]	NA	NA	81 (29.89)	1 [Reference]	NA	NA
5-9	239 (28.15)	1.63 (1.31-2.03)	<.001	7.2	86 (31.73)	1.95 (1.36-2.80)	<.001	10.9
10-14	214 (25.21)	1.48 (1.19-1.85)	<.001	NC	68 (25.09)	1.80 (1.23-2.63)	<.001	NC
15-20	122 (14.37)	1.16 (0.88-1.52)	.29	NC	34 (12.55)	1.50 (0.93-2.43)	.10	NC
20-24	7 (0.82)	1.22 (0.51-2.95)	.65	NC	2 (0.74)	1.69 (0.36-8.03)	.51	NC
Body mass index^b^								
Normal, 18.5-24.9	30 (3.53)	1 [Reference]	NA	NA	30 (11.07)	1 [Reference]	NA	NA
Underweight, <18.5	294 (34.63)	1.13 (0.70-1.84)	.61	NC	90 (33.21)	3.95 (2.28-6.85)	<.001	7.0
Overweight, 25.0-29.9	231 (27.21)	0.83 (0.67-1.02)	.08	NC	76 (28.04)	0.83 (0.58-1.19)	.31	NC
Obesity, ≥30.0	294 (34.63)	0.84 (0.69-1.04)	.10	NC	75 (27.68)	0.66 (0.46-0.95)	.03	NC
Cranial radiotherapy								
None	371 (44.06)	1 [Reference]	NA	NA	78 (29.66)	1 [Reference]	NA	NA
>0 and <30 Gy	348 (41.33)	1.15 (0.84-1.57)	.38	NC	85 (32.32)	1.62 (0.92-2.86)	.09	NC
≥30 Gy	123 (14.61)	1.75 (1.41-2.18)	<.001	5.4	100 (38.02)	5.22 (3.74-7.30)	<.001	33.0
Testicular or pelvic irradiation								
None	661 (77.86)	1 [Reference]	NA	NA	172 (63.47)	1 [Reference]	NA	NA
Yes	188 (22.14)	1.17 (0.92-1.47)	.20	NC	99 (36.53)	1.70 (1.19-2.44)	<.001	11.5
Alkylating agents, mg/m^2^								
0	370 (43.84)	1 [Reference]	NA	NA	114 (42.22)	1 [Reference]	NA	NA
>0 to <4000	114 (13.51)	1.13 (0.86-1.50)	.38	NC	33 (12.22)	1.05 (0.63-1.75)	.84	NC
≥4000 to <8000	134 (15.88)	1.07 (0.83-1.37)	.60	NC	39 (14.44)	1.19 (0.76-1.85)	.44	NC
≥8000	226 (26.78)	1.11 (0.88-1.39)	.37	NC	84 (31.11)	1.09 (0.74-1.59)	.67	NC
Glucocorticoid dose, mg/m^2^								
0	421 (50.42)	1 [Reference]	NA	NA	171 (63.81)	1 [Reference]	NA	NA
>0 to <3240	153 (18.32)	0.93 (0.73-1.17)	.53	NC	32 (11.94)	0.57 (0.36-0.89)	.01	NC
≥3240	261 (31.26)	1.34 (1.09-1.66)	<.001	5.9	65 (24.25)	1.04 (0.72-1.49)	.85	NC
Hematopoietic stem cell transplant								
No	760 (89.52)	1 [Reference]	NA	NA	223 (82.29)	1 [Reference]	NA	NA
Yes	89 (10.48)	1.38 (0.97-1.98)	.07	NC	48 (17.71)	1.06 (0.62-1.81)	.83	NC
Smoking								
Never	533 (65.00)	1 [Reference]	NA	NA	185 (70.88)	1 [Reference]	NA	NA
Previous	83 (10.12)	1.05 (0.80-1.39)	.71	NC	19 (7.28)	1.02 (0.61-1.73)	.93	NC
Current	204 (24.88)	1.55 (1.27-1.90)	<.001	6.1	57 (21.84)	1.71 (1.21-2.43)	<.001	6.7
Sedentary behavior								
No	648 (81.51)	1 [Reference]	NA	NA	185 (72.83)	1 [Reference]	NA	NA
Yes	147 (18.49)	0.95 (0.62-1.46)	.83	NC	69 (27.17)	1.91 (1.10-3.33)	.02	3.2

Abbreviations: AF, attributable fractions; BMD, bone mineral density; NA, not applicable; NC, not calculated; OR, odds ratio.

^a^ Models are adjusted for age at assessment and sex.

^b^ Body mass index is calculated as weight in kilograms divided by height in meters squared.

Compared with those with normal BMD, survivors with severe deficits were more likely to be aged 5 to 9 years at diagnosis, to be exposed to 30 Gy or greater CRT (OR, 5.22; 95% CI, 3.74-7.30; AF, 33.0%), to be exposed to testicular or pelvic radiation (OR, 1.70, 95% CI, 1.19-2.44; AF, 11.5%), to have underweight (OR, 3.95; 95% CI, 2.28-6.85; AF, 7.0%), to be sedentary (OR, 2.06, 95% CI, 1.15-3.69; AF, 6.2%), or to be currently smoking (OR, 1.71, 95% CI, 1.21-2.43; AF, 6.7%). These variables jointly had an AF of 72.3% for the 271 survivors with severe BMD, with 55.4% for age at diagnosis and exposure to 30 Gy or greater CRT or 3240 mg/m^2^ or greater of glucocorticoids and 9.9% for current smoking and sedentary behavior. (Table 3).

### Comorbid Conditions, Substance Use, Activity, Diet, and BMD

These variables had a joint AF of 28.1% for the 716 survivors with moderate BMD deficit in this model focused on comorbid conditions, with 10.2% for hypogonadism and GHD and 7.0% for current smoking (Table 4). Compared with those with normal BMD, survivors with severe deficits were more likely to have hypogonadism (untreated in 195 of 336 female participants [58.0%] and 164 of 309 male participants [53.1%]) or GHD (untreated in 387 of 407 survivors [95.1%]), be aged 5 to 9 years at diagnosis, to have underweight, or to be sedentary. These variables jointly had an AF of 79.1% for the 237 survivors with severe BMD deficit, with 51.1% for hypogonadism and GHD (Table 4).

**Table 4.** Comorbid Conditions, Lifestyle, and Moderate and Severe BMD Deficit Risk at Most Recent BMD Evaluation^a^

Factor	BMD deficit							
	Moderate (n = 716)				Severe (n = 237)			
	No. (%)	OR (95% CI)	*P* value	AF, %	No. (%)	OR (95% CI)	*P* value	AF, %
Race/ethnicity								
Non-Hispanic White	645 (90.1)	1 [Reference]	NA	NA	215 (90.7)	1 [Reference]	NA	NA
Hispanic	23 (3.2)	0.89 (0.54-1.49)	.66	NC	12 (5.1)	1.03 (0.50-2.15)	.93	NC
Non-Hispanic Black	36 (5.0)	0.20 (0.14-0.30)	<.001	NC	7 (3.0)	0.10 (0.04-0.25)	<.001	NC
Additional groups	12 (1.7)	0.84 (0.42-1.66)	.61	NC	3 (1.3)	0.52 (0.15-1.84)	.31	NC
Age at diagnosis, y								
0-4	214 (29.9)	1 [Reference]	NA	NA	68 (28.7)	1 [Reference]	NA	NA
5-9	207 (28.9)	1.78 (1.42-2.25)	<.001	10.9	76 (32.1)	2.00 (1.37-2.92)	<.001	13.3
10-14	184 (25.7)	1.40 (1.10-1.77)	<.001	NC	60 (25.3)	1.54 (1.04-2.29)	.03	NC
15-19	106 (14.8)	1.08 (0.82-1.42)	.58	NC	31 (13.1)	1.25 (0.78-2.01)	.36	NC
20-24	5 (0.7)	0.68 (0.23-2.00)	.48	NC	2 (0.8)	1.24 (0.27-5.64)	.78	NC
Body mass index^b^								
Normal, 18.5-24.9	253 (35.3)	1 [Reference]	NA	NA	78 (32.9)	1 [Reference]	NA	NA
Underweight, <18.5	25 (3.5)	1.10 (0.66-1.83)	.71	NC	25 (10.5)	3.21 (1.79-5.73)	<.001	8.5
Overweight, 25.0-29.9	194 (27.1)	0.73 (0.58-0.91)	<.001	NC	66 (27.8)	0.81 (0.56-1.17)	.26	NC
Obesity, ≥30.0	244 (34.1)	0.72 (0.57-0.90)	<.001	NC	68 (28.7)	0.64 (0.43-0.94)	.02	NC
Hypogonadism								
No	567 (79.2)	1 [Reference]	NA	NA	140 (59.1)	1 [Reference]	NA	NA
Yes	149 (20.8)	1.56 (1.23-1.97)	<.001	5.3	97 (40.9)	3.27 (2.35-4.55)	<.001	25.1
Growth hormone deficiency								
No	610 (85.2)	1 [Reference]	NA	NA	153 (64.6)	1 [Reference]	NA	NA
Yes	106 (14.8)	1.95 (1.46-2.60)	<.001	4.9	84 (35.4)	5.28 (3.68-7.56)	<.001	26.0
Vitamin D status, ng/mL								
Normal (≥30)	261 (36.5)	1 [Reference]	NA	NA	108 (45.6)	1 [Reference]	NA	NA
Insufficient (<30 and ≥20)	276 (38.5)	1.17 (0.95-1.44)	.14	NC	79 (33.3)	0.94 (0.67-1.31)	.73	NC
Deficient (<20)	179 (25.0)	1.18 (0.93-1.50)	.17	NC	50 (21.1)	0.89 (0.60-1.33)	.58	NC
Smoking status								
Never	477 (66.6)	1 [Reference]	NA	NA	171 (72.2)	1 [Reference]	NA	NA
Previous	76 (10.6)	1.03 (0.77-1.38)	.83	NC	19 (8.0)	0.82 (0.49-1.38)	.46	NC
Current	163 (22.8)	1.44 (1.16-1.80)	<.001	7.0	47 (19.8)	1.43 (0.99-2.08)	.06	NC
Sedentary behavior								
No	688 (96.1)	1 [Reference]	NA	NA	218 (92.0)	1 [Reference]	NA	NA
Yes	28 (3.9)	0.99 (0.63-1.57)	.97	NC	19 (8.0)	2.06 (1.15-3.69)	.01	6.2

Abbreviations: AF, attributable fractions; BMD, bone mineral density; NA, not applicable; NC, not calculated; OR, odds ratio.

SI conversion factor: To convert vitamin D to nanomoles per liter, multiply by 2.496.

^a^ Models are adjusted for age at assessment, sex, hypertension, and high-density lipoprotein levels.

^b^ Body mass index is calculated as weight in kilograms divided by height in meters squared.

### Association of BMD Deficits at Most-Recent BMD Evaluation With Long-Term Outcomes

The prevalence rates of long-term outcomes are reported by BMD status in eTable 1 in Supplement 1. Compared with those with normal BMD, survivors with deficits were less likely to live alone or be employed. Survivors with deficits were more likely to require personal care assistance, report depressive symptoms, or report poor QOL on bodily pain, physical function, role physical, and general health SF-36 subscales (Table 5; eTable 2 in Supplement 1). Survivors with moderate deficits were more likely to report poor QOL on social function, role emotional, and mental health SF-36 subscales (eTable 2 in Supplement 1).

**Table 5.** Moderate and Severe BMD Deficits at Most Recent BMD Evaluation and Social and Functional Independence, HRQoL, and Depression^a^

Measure	BMD Status		
	Normal (n = 2799)	Moderate (n = 849)	Severe (n = 271)
**Independent living^b^**			
No./total No. (%)	1989/2673 (74.4)I	540/810 (66.7)	121/253 (47.8)
OR (95% CI)	1 [Reference]	0.76 (0.62-0.92)	0.47 (0.35-0.65)
*P* value	NA	.006	<.001
**Employment^c^**			
No./total No. (%)	1892/2714 (69.7)	550/823 (66.8)	151/255 (59.2)
OR (95% CI)	1 [Reference]	0.79 (0.66-0.94)	0.65 (0.49-0.86)
*P* value	NA	.01	.003
**Assistance with personal needs** ^d^			
No./total No. (%)	73/2668 (2.8)	37/810 (4.6)	15/250 (6.0)
OR (95% CI)	1 [Reference]	1.68 (1.09-2.57)	1.89 (1.01-3.52)
*P* value	NA	.02	.046
**Education** ^e^			
No./total No. (%)	1667/2485 (67.1)	484/764 (63.4)	144/234 (61.5)
OR (95% CI)	1 [Reference]	0.87 (0.73-1.04)	0.92 (0.68-1.23)
*P* value	NA	.13	.56
**Physical component summary**			
No./total No. (%)	425/2592 (16.4)	153/790 (19.4)	64/243 (26.3)
OR (95% CI)	1 [Reference]	1.48 (1.18-1.84)	2.57 (1.83-3.60)
*P* value	NA	<.001	<.001
**Mental component summary**			
No./total No. (%)	536/2590 (20.7)	173/790 (21.9)	50/243 (20.6)
OR (95% CI)	1 [Reference]	1.23 (1.00-1.51)	1.20 (0.85-1.69)
*P* value	NA	.045	.31
**Depression**			
No./total No. (%)	340/2676 (12.7)	143/809 (17.7)	41/251 (16.3)
OR (95% CI)	1 [Reference]	1.42 (1.13-1.77)	1.29 (0.89-1.88)
*P* value	NA	.002	.18

Abbreviations: BMD, bone mineral density; HRQoL, health-related quality of life; NA, not applicable; OR, odds ratio.

^a^ Model was adjusted for sex, race and ethnicity, age at diagnosis, age at first assessment, diagnosis, and receipt of cranial radiotherapy.

^b^ Model probability of living independently.

^c^ Model probability of having full-time or part-time employment.

^d^ Model probability of needing assistance with personal care needs.

^e^ Model probability of having a college or university education or vocational training after high school.

### Association of Treatment Exposures With BMD Status Change

Among survivors with normal BMD at baseline, treatment and demographic parameters associated with developing deficits were 30 Gy or greater CRT (OR, 2.94, 95% CI, 1.46-5.91; AF, 8.8%). Non-Hispanic Black participants and those who belonged to the additional race and ethnicity groups in our study were less like to have BMD status change (OR, 0.33; 95% CI, 0.15-0.71; AF, 14.5%). These characteristics had a joint AF of 23.3% among the 95 survivors whose BMD status declined (eTable 3 in Supplement 1).

### Association of Comorbid Conditions and BMD Status Change

Among survivors with normal BMD at baseline, there were no comorbid conditions associated with developing deficits. In this model, the AF for non-Hispanic Black participants and participants from the additional racial groups in our study was 14.2% among the 95 survivors whose BMD status declined (eTable 4 in Supplement 1).

## Discussion

Our comprehensive evaluation of BMD status among adult survivors of childhood cancer found that 22% and 7% of survivors have moderate or severe deficits decades after treatment has ended. Survivors exposed to 30 Gy or greater of CRT with normal BMD remained at greatest risk for new onset of moderate and severe deficits during survivorship. In this study, BMD deficits appeared to be jointly attributed to cancer treatment, comorbid conditions, smoking, and sedentary behavior. Because these deficits increase risk for loss of social and functional independence, depressive symptoms, and poor QOL, interventions that either treat comorbid conditions or that target health behaviors are warranted.

Our findings that risk for BMD deficits are greatest among survivors exposed to high-dose glucocorticoids or cranial radiation, those who have GHD or hypogonadism, and those who smoke or are sedentary are consistent with the International Late Effects of Childhood Cancer Guideline Harmonization Group’s (IGHG) recent report on BMD among childhood, adolescent, and young adult cancer survivors.^37^ This study further filled in gaps identified by the IGHG, showing that testicular or pelvic radiation and glucocorticoids were associated with BMD well into survivorship. Our data reinforce the Children’s Oncology Group (COG) guidelines that recommend screening at-risk childhood cancer survivors upon entry to long-term follow-up with repeat BMD evaluations.^38^

By age 45 years, 95% of childhood cancer survivors have at least 1 comorbid condition^4^; 50% have endocrinopathies likely disrupting bone health.^39^ In our study, 10% of moderate and 51% of severe BMD deficits were attributable to hypogonadism and GHD, respectively, both treatable with hormone replacement.^40^ More direct interventions for bone loss include bisphosphonates,^41^ routinely prescribed for osteoporosis,^42^ even in children,^43^ and denosumab. Bisphosphonates inhibit the function^44^ and promote apoptosis of osteoclasts,^45^ bone cells responsible for bone resorption. Denosumab, a RANKL inhibitor interfering with osteoclast-mediated bone resorption and turnover,^46^ is used to treat BMD loss in adults^47^ and patients with cancer.^48^ Nonrandomized studies using bisphosphonates (pamidronate, alendronate) or denosumab, conducted during and after the completion of chemotherapy in children, show improvement in BMD.^49,50,51,52,53,54,55^ Because the indirect benefits of hormone replacement on BMD outcomes among childhood cancer survivors are unclear^56^ and because bisphosphonates and denosumab have not been evaluated in randomized trials, additional studies are needed to strengthen support for the use of pharmaceuticals to treat BMD in children during treatment and among survivors of childhood cancer.^57,58,59,60^

In addition to treating comorbid conditions, modifying health behaviors has the potential to improve survivor’s bone health. Smoking increased the odds for BMD deficits by 40% to 70% in our study. Even though survivors are less likely to smoke than peers,^61^ the significance of not smoking should be emphasized, particularly among survivors exposed to cranial radiation, as they are least likely to quit.^61^ Unfortunately, traditional smoking cessation interventions have had limited success^62^ with only 1 study reporting effective cessation rates with peer-delivered phone counselling.^63,64^ More effective strategies are needed.

Exercise, specifically weight-bearing and plyometric movements, positively impacts bone quality. Several studies support the association between physical activity in childhood with adult bone density.^65,66^ Unfortunately, children on-therapy are often sedentary.^67,68^ Interventions designed to prevent bone loss during cancer treatment have been unsuccessful,^69,70^ suggesting that the impact of treatment on bone metabolism is too great to overcome with exercise alone. Similar to children with cancer, survivors do not engage in regular physical activity^71,72^ and are less likely to participate in bone-beneficial exercise such as resistance training.^73^ Exercise interventions to improve bone health in survivors have had only moderate success,^74^ with some results suggesting marginal benefits among survivors with worse BMD.^75^ However, low-magnitude (<1.0 g) mechanical stimulation, which exposes bone to mechanical signals similar to those elicited from high-impact loading physical activity, improves total body BMD, tibial trabecular bone volume, and bone metabolism among child and adolescent survivors of cancer.^23^ We previously conducted a prospective, double-blind, placebo-controlled trial among 48 survivors 5 or more years after diagnosis,^24^ in which participants stood on a vibrating platform for 10 minutes, twice a day for 1 year. This intervention was noninvasive and nonpharmacologic^76^ and may be an attractive preventive therapy for bone loss during childhood cancer treatment^77^ or a potential intervention in long-term survivors who have moderate or severe BMD deficits.

We did not find an association between self-reported dietary intake and BMD in our population. However, self-reported diet does not capture the true nutritional status of an individual. Measures of albumin, prealbumin, hemoglobin, and total cholesterol levels are useful biomarkers of malnutrition in older adults.^78^ Given that this cohort continues to be followed up prospectively and is available for intervention, future work to document nutritional status and to remediate nutritional deficits may improve bone health. While studies evaluating dietary counseling or education with or without vitamin D and calcium supplementation do not show efficacy to improve BMD in childhood cancer survivors, the quality of this evidence is low.^79^ Meta-analyses of randomized trials indicate that adding vitamin K to calcium and/or vitamin D supplementation^80,81^ has a positive effect on lumbar BMD in adults with osteoporosis. In addition, observational studies show that intakes consistent with low dietary inflammatory indices,^82,83^ Mediterranean dietary patterns,^84^ and higher protein content are associated with higher BMD.^85^

Overall, our risk factors associated with BMD deficits were consistent with those of IGHG,^37^ highlighting that children exposed to CRT, testicular or pelvic radiation, and glucocorticoids or those at risk for GHD or hypogonadism had the greatest risk for BMD deficits. Survivors should be screened for BMD deficits during follow-up and counselled to optimize health behaviors to minimize progression of bone loss. Appropriate counselling is particularly important for survivors as they enter adulthood, moving from pediatric to adult health care systems when primary care clinicians are largely responsible for managing their health care. Clinicians will have few survivors in their practices and may not be aware of surveillance guidelines for this vulnerable population. In addition to further randomized clinical trials evaluating the efficacy of hormone and drug interventions, alone or in combination with lifestyle modifications, creative practical approaches to educate both survivors and clinicians are needed.

### Limitations

These results should be interpreted in the context of limitations. First, our cohort consisted primarily of non-Hispanic White and Black participants, thus lacking a comprehensive evaluation of BMD outcomes in individuals of other ethnic and racial groups. Furthermore, all eligible survivors were not included in analyses. If those with poor bone health were more or less likely to participate, our results may overestimate or underestimate the prevalence, severity, and factors attributing to BMD deficits. However, all SJLIFE participants are offered BMD screening independently of their treatment history, providing a comprehensive view of bone health status across treatment exposure. Second, our clinical assessment did not include trauma history or spine radiographs for every participant; we were unable to evaluate prevalent scoliosis, prolonged periods of immobilization, or traumatic fractures as risk factors for low BMD. Third, remote treatment-related risk factors including severe malnutrition, inactivity during childhood, and significant bone loss during cancer therapy were not evaluated; these factors may influence long-term BMD outcomes. Fourth, many of our participants had only 1 QCT assessment available. Thus, cross-sectional evaluation limits our ability to know how modifiable health behaviors contribute to BMD deficits. We captured BMD status change among a subpopulation of our participants, showing that new or more severe deficits can develop within 3 to 5 years. However, it is unlikely that these deficits occur in the absence of new or worsening comorbid conditions, other than endocrinopathies. Given that chronic conditions and aging are associated with chronic systemic inflammation, inflammation and the pathologies of the comorbid conditions likely influence bone metabolism, contributing to the progressive declines in bone health.^86,87^ Additional research is needed to determine the interplay between these conditions and declining bone health in this population. Collectively, our models explained some of the moderate and most of the severe BMD deficit cases in our cohort, attributing deficits to treatment exposures, comorbid conditions, smoking, and sedentary behavior.

Unexplained cases in our cohort may be partially attributed to genetics, as 80% of peak bone density^88^ and 50% to 90% of bone mass and quality can be explained by genetics, with a large body of evidence demonstrating the strong influence of heritability on BMD variation.^89,90,91,92,93,94,95^ A recent study revealed a locus with sex- and therapy-specific fracture risk effects in childhood cancer, suggesting a genetic influence of bone resorption pathways in female survivors.^96^ We did not account for genetic factors in this analysis. Future research should incorporate genetic risk for bone morbidities and treatment-genetic interactions so that individuals who may benefit from targeted interventions can be identified early.

## Conclusions

In this cohort study, moderate or severe BMD was prevalent in survivors of childhood cancer. CRT, testicular or pelvic radiation, hypogonadism, GHD, smoking, and sedentary behavior were associated with increased risk of severe BMD deficits. Future research should examine modifiable risk factors (hypogonadism, GHD, smoking, and sedentary behavior) for possible targeted interventions.

## Notes

Supplement 1. **eFigure 1.** Diagram Illustrating the Flow of Participation in Analysis **eFigure 2.** Proportion of Participants With BMD Deficits at Most Recent BMD Evaluation **eFigure 3.** Proportion of Participants With BMD Deficits at Most Recent Bone Evaluation, by Primary Cancer Diagnosis **eTable 1.** Association of Long-Term Outcomes by BMD Status at Most Recent Evaluation **eTable 2.** Association of Moderate and Severe BMD Deficits at Most Recent Evaluation With Individual Components of HRQoL Summary Scores **eTable 3.** Association of Treatment Exposures With BMD Decline, Among Participants With Normal BMD at First BMD Evaluation **eTable 4.** Association of Comorbid Conditions With BMD Decline, Among Participants With Normal BMD at First BMD Evaluation

Supplement 2.
Data Sharing Statement
