# Supplementary material for: Attributable Risk and Consequences of Bone Mineral Density Deficits in Childhood Cancer Survivors
Source: JAMA Netw Open. 2025 Jan 10;8(1):e2454069. doi: 10.1001/jamanetworkopen.2024.54069 (PMC11724346; doi:10.1001/jamanetworkopen.2024.54069)
Supplement: Supplement 1. — eFigure 1. Diagram Illustrating the Flow of Participation in Analysis eFigure 2. Proportion of Participants With BMD Deficits at Most Recent BMD Evaluation eFigure 3. Proportion of Participants With BMD Deficits at Most Recent Bone Evaluation, by Primary Cancer Diagnosis eTable 1. Association of Long-Term Outcomes by BMD Status at Most Recent Evaluation eTable 2. Association of Moderate and Severe BMD Deficits at Most Recent Evaluation With Individual Components of HRQoL Summary Scores eTable 3. Association of Treatment Exposures With BMD Decline, Among Participants With Normal BMD at First BMD Evaluation eTable 4. Association of Comorbid Conditions With BMD Decline, Among Participants With Normal BMD at First BMD Evaluation [file jamanetwopen-e2454069-s001.pdf]

Supplemental Online Content

Goodenough CG, Baedke JL, Delaney AM, et al. Attributable risk and consequences of bone mineral density deficits in childhood cancer survivors. *JAMA Netw Open.* 2025;8(1):e2454069. doi:10.1001/jamanetworkopen.2024.54069

eFigure 1. Diagram Illustrating the Flow of Participation in Analysis

eFigure 2. Proportion of Participants With BMD Deficits at Most Recent BMD Evaluation

eFigure 3. Proportion of Participants With BMD Deficits at Most Recent Bone Evaluation, by Primary Cancer Diagnosis

eTable 1. Association of Long-Term Outcomes by BMD Status at Most Recent Evaluation

eTable 2. Association of Moderate and Severe BMD Deficits at Most Recent Evaluation With Individual Components of HRQoL Summary Scores

eTable 3. Association of Treatment Exposures With BMD Decline, Among Participants With Normal BMD at First BMD Evaluation

eTable 4. Association of Comorbid Conditions With BMD Decline, Among Participants With Normal BMD at First BMD Evaluation

This supplemental material has been provided by the authors to give readers additional information about their work.

© 2025 Goodenough CG et al. *JAMA Network Open*.

eFigure 1. Diagram Illustrating the Flow of Participation in Analysis

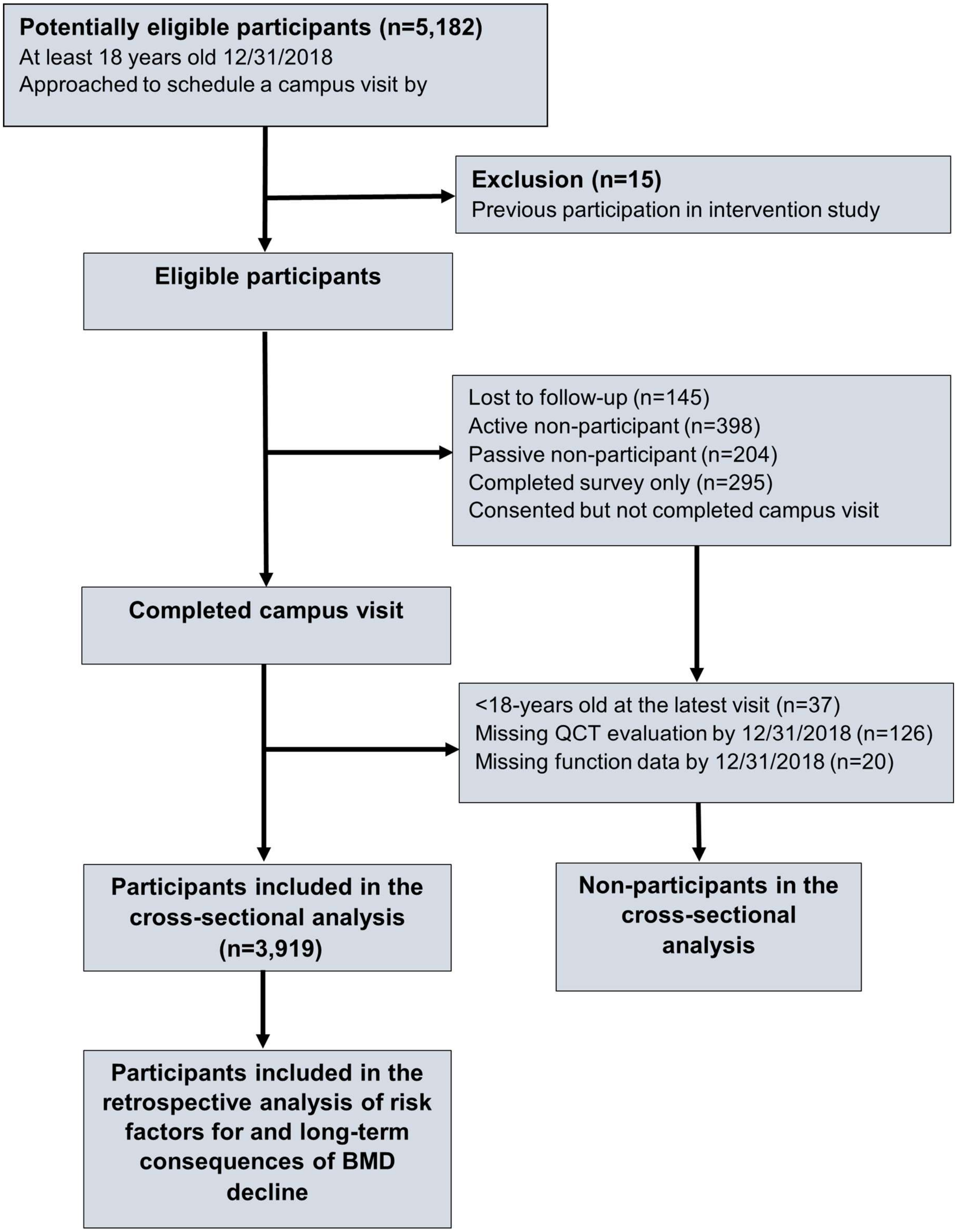

(BMD: bone mineral density; n: number; QCT: quantitative computed tomography)

© 2025 Goodenough CG et al. *JAMA Network Open*.

eFigure 2. Proportion of Participants With BMD Deficits at Most Recent BMD Evaluation

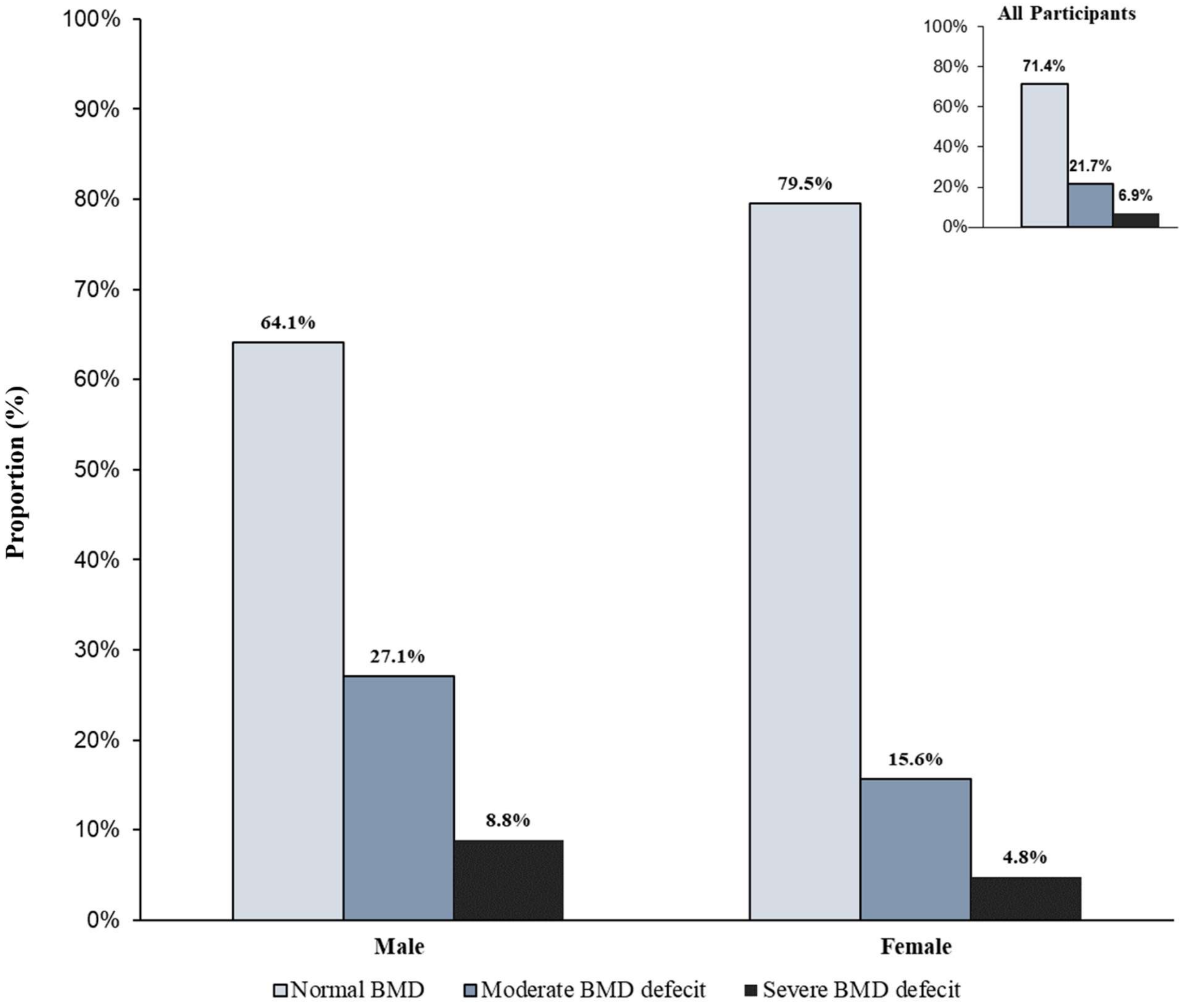

BMD: bone mineral density; %: percent

© 2025 Goodenough CG et al. *JAMA Network Open*.

eFigure 3. Proportion of Participants With BMD Deficits at Most Recent Bone Evaluation, by Primary Cancer Diagnosis

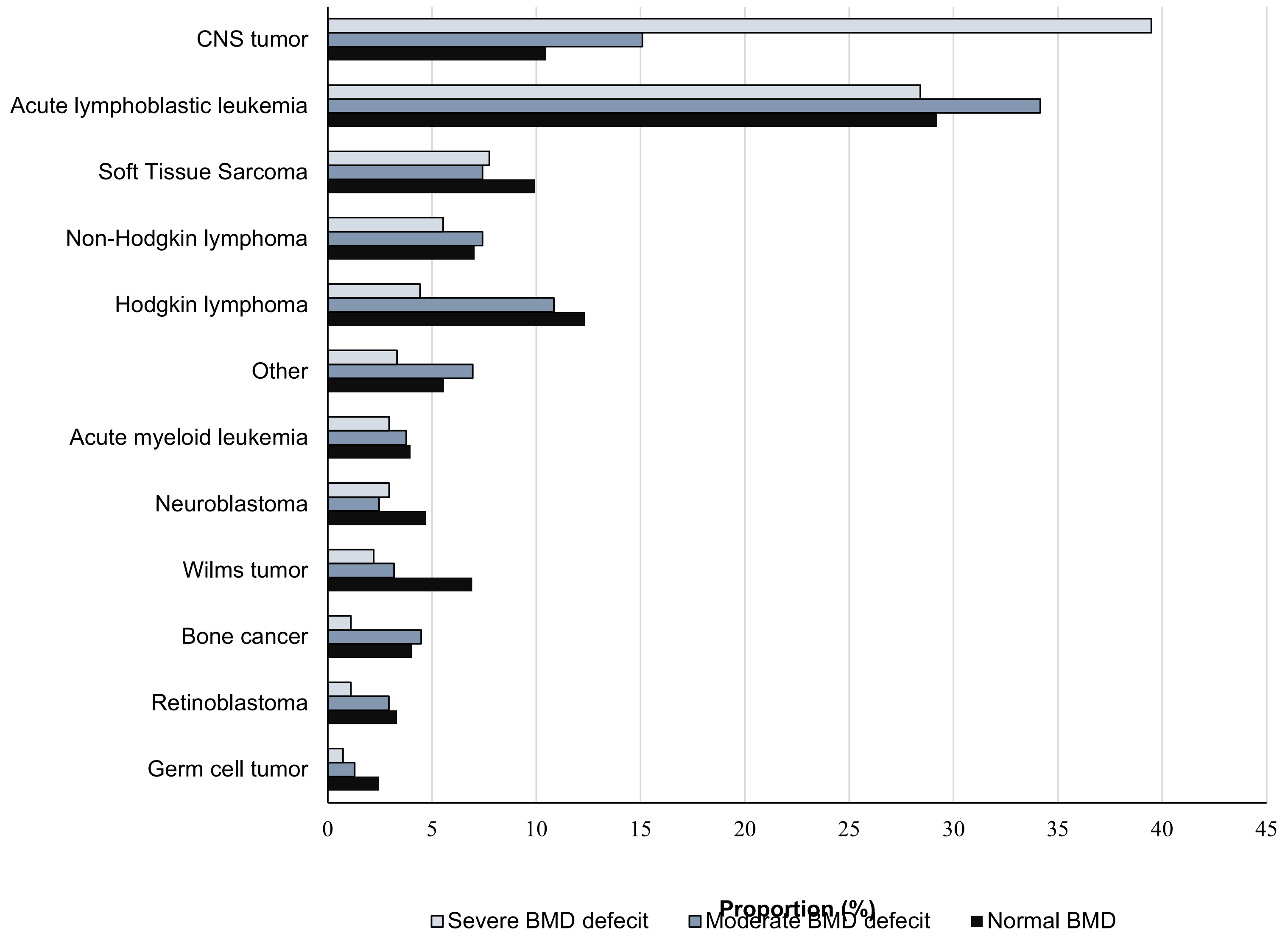

Abbreviations; BMD: bone mineral density; n: number; QCT: quantitative computed tomography; CNS: central nervous system

© 2025 Goodenough CG et al. *JAMA Network Open*.

eTable 1. Association of Long-Term Outcomes by BMD Status at Most Recent Evaluation

Long-term Outcome, No. (%)	BMD Deficit			
	**Normal (n=2,799)**	**Moderate (n=849)**	**Severe (n=271)**	***p***
**Functional and Social Independence**				
**Assistance with personal needs**				<0.01
No	2,616 (93·5)	780 (91·9)	15 (5·5)	
Yes	77 (2·8)	37 (4·4)	243 (89·7)	
Unknown	106 (3·8)	32 (3·8)	13 (4·8)	
**Educational Attainment**				0·01
<High school	232 (8·3)	92 (10·8)	19 (7·0)	
High school graduate	594 (21·2)	192 (22·6)	75 (27·7)	
College graduate or vocational training	1,680 (60·0)	487 (57·4)	147 (54·2)	
Unknown	293 (10·5)	78 (9·2)	30 (11·1)	
**Employment Status**				<0·01
Never employed	154 (5·5)	61 (7·2)	46 (17·0)	
Unemployed	680 (24·3)	213 (25·1)	62 (22·9)	
Part-time	355 (12·7)	116 (13·7)	40 (14·8)	
Full-time	1,549 (55·3)	440 (51·8)	115 (42·4)	
Unknown	61 (2·2)	19 (2·2)	8 (3·0)	
**Independent Living**				<0·01
Yes (lives with spouse/partner or alone)	2,002 (71·5)	544 (64·1)	125 (46·1)	
No (lives with parents or other; relatives (not including minor children))	694 (24·8)	273 (32·2)	136 (50·2)	
Unknown	103 (3·7)	32 (3·8)	10 (3·7)	
**Body Pain**				0·36
No	2,202 (78·7)	661 (77·9)	202 (74·5)	
Yes	484 (17·3)	156 (18·4)	55 (20·3)	
Unknown	113 (4·0)	32 (3·8)	14 (5·2)	
**Depression**				<0·01
No	2,356 (84·2)	672 (79·2)	217 (80·1)	
Yes	343 (12·3)	144 (17·0)	42 (15·5)	
Unknown	100 (3·6)	33 (3·9)	12 (4·4)	
**Health Related Quality of Life (HRQoL)**				
**Physical**				<0·01
Non-Reduced	2,185 (78·1)	644 (75·9)	183 (67·5)	
Reduced	428 (15·3)	153 (18·0)	67 (24·7)	
Unknown	186 (6·6)	52 (6·1)	21 (7·7)	
**Mental**				0·82
Non-Reduced	2,070 (74·0)	624 (73·5)	199 (73·4)	
Reduced	541 (19·3)	173 (20·4)	51 (18·8)	
Unknown	188 (6·7)	52 (6·1)	21 (7·7)	

Notes: Values are column percents. Unknown indicates those who did not respond to the corresponding questions on the self-report questionnaire.

Abbreviations: HRQoL: health-related quality of life; <: less than; n: number; p: probability

© 2025 Goodenough CG et al. *JAMA Network Open*.

eTable 2. Association of Moderate and Severe BMD Deficits at Most Recent Evaluation With Individual Components of HRQoL Summary Scores

BMD Status	No.	Bodily Pain			No.	Physical Function		
		Row %	OR (95% CI)	p		Row %	OR (95% CI)	p
Normal	2,664	18·1	Ref		2,662	15·9	Ref	
Moderate	810	19·3	1·28 (1·03–1·58)	0·024	808	18·2	1·47 (1·17–1·84)	<·001
Severe	250	21·2	1·71 (1·21–2·42)	0·002	249	25·7	2·47 (1·76–3·48)	<·001

BMD Status	No.	Role Physical			No.	General Health		
		Row %	OR (95% CI)	p		Row %	OR (95 % CI)	p
Normal	2,654	17·6	Ref		2,658	26·1	Ref	
Moderate	807	22·3	1·59 (1·29–1·96)	<·001	809	27·9	1·26 (1·04–1·52)	0·017
Severe	250	24·0	1·70 (1·21–2·38)	0·002	249	31·3	1·64 (1·21–2·22)	0·002

BMD Status	No.	Social Function			No.	Role Emotional		
		Row %	OR (95% CI)	p		Row %	OR (95% CI)	p
Normal	2,687	17·6	Ref		2,644	16·5	Ref	
Moderate	817	19·3	1·28 (1·03–1·58)	0·023	806	18·4	1·33 (1·07–1·65)	0·010
Severe	251	17·9	1·15 (0·81–1·65)	0·432	249	18·5	1·36 (0·95–1·94)	0·095

BMD Status	No.	Mental Health		
		Row %	OR (95% CI)	p
Normal	2,661	21·2	Ref	
Moderate	808	24·1	1·35 (1·11–1·64)	0·003
Severe	247	20·6	1·16 (0·83–1·63)	0·383

Note: Model was adjusted for sex, race, age at diagnosis, age at first assessment, diagnosis, CRT. Odds ratio comparing prevalence rates.

Row %= Among people with a particular BMD status, the percentage of people with the event for a particular outcome variable

Abbreviations: BMD: bone mineral density; CI: confidence interval; <: less than; n: number; OR: odds ratio: %: percent; p: probability; Ref: reference

© 2025 Goodenough CG et al. *JAMA Network Open*.

eTable 3. Association of Treatment Exposures With BMD Decline, Among Participants With Normal BMD at First BMD Evaluation

Parameter	No.	Row %	OR	95% CI	p	AF (%)
**Age at cancer diagnosis (years)**						
0–4	440	6·8	1·00	Ref	0·40	
5–9	276	11·2	1·57	(0·90, 2·74)		
10–14	281	8·2	1·15	(0·636, 2·09)		
>15	203	6·9	1·01	(0·51, 2·01)		
**Race/ethnicity**						
White-non-Hispanic	952	9·4	1·00	Ref	0·00	
Non-White non-Hispanic	248	3·2	0·33	(0·15, 0·71)		14·5
**Sex**						
Male	553	9·0	Ref	Ref	0·51	
Female	647	7·4	0·86	(0·55, 1·35)		
**Brain radiation dose**						
None	847	8·1	1·00	Ref	<0·01	
>0 and <30Gy	274	5·5	0·57	(0·29, 1·13)		
≥30Gy	79	19·0	2·94	(1·46, 5·91)		8·8
**Testicular or pelvic radiation**						
No	919	8·4	1·00	Ref	0·26	
Yes	183	7·1	0·69	(0·36, 1·32)		
**Corticosteroid (mg/m2)**						
None	574	8·9	1·00	Ref	0·01	
>0 and <3240	305	4·3	0·60	(0·30, 1·18)		
≥3240	321	10·6	1·69	(0·99, 2·9)		

Note: Model is adjusted for time between QCTs, smoking status, physical activity, vitamin A intake, and caffeine intake.

AF: attributable fractions; BMD: bone mineral density; CI: confidence interval; <: less than; >: more than; ≥: more than or equal to; n: number; OR: odds ratio; %: percent; p: probability; Ref: reference

© 2025 Goodenough CG et al. *JAMA Network Open*.

eTable 4. Association of Comorbid Conditions With BMD Decline, Among Participants With Normal BMD at First BMD Evaluation

Parameter	No.	Row %	OR	95% CI	*p*	AF (%)
**Age at cancer diagnosis (years)**						
0–4	440	6·8	1·00	Ref	0·20	
5–9	276	11·2	1·74	(1·006, 3·002)		
10–14	281	8·2	1·20	(0·665, 2·158)		
>15	203	6·9	0·98	(0·499, 1·93)		
**Race/ethnicity**						
White-non-Hispanic	952	9·4	1·00	Ref	0·01	
Non-White non-Hispanic	248	3·2	0·34	(0·161, 0·734)		14·2
**Sex**						
Male	553	9·0	1·00	Ref	0·59	
Female	647	7·4	0·89	(0·57, 1·374)		
**Hypogonadism**						
No	1,013	7·7	1·00	Ref	0·11	
Yes	187	10·7	1·58	(0·896, 2·781)		
**Growth Hormone Deficiency**						
No	1,028	8·2	1·00	Ref	0·46	
Yes	74	7·5	0·71	(0·283, 1·772)		

Note: Model is adjusted for time between QCTs, smoking status, physical activity, vitamin A intake, and caffeine intake.

AF: attributable fractions; BMD: bone mineral density; CI: confidence interval; >: more than; n: number; OR: odds ratio; %: percent; p: probability; Ref: reference

© 2025 Goodenough CG et al. *JAMA Network Open*.
