# Supplementary material for: Attributable Risk and Consequences of Bone Mineral Density Deficits in Childhood Cancer Survivors
Source: JAMA Netw Open. 2025 Jan 10;8(1):e2454069. doi: 10.1001/jamanetworkopen.2024.54069 (PMC11724346; doi:10.1001/jamanetworkopen.2024.54069)
Supplement: Supplement 2. — Data Sharing Statement [file jamanetwopen-e2454069-s002.pdf]

Data Sharing Statement

Goodenough. Attributable Risk and Consequences of Bone Mineral Density Deficits in Childhood Cancer Survivors. *JAMA Netw Open*. Published January 10, 2025. doi:10.1001/jamanetworkopen.2024.54069

Data
Data available: Yes
Data types: Deidentified participant data
How to access data: https://zenodo.org/
When available: With publication

Supporting Documents
Document types: None

Additional Information
Who can access the data: The data will be available in the public domain.
Types of analyses: The data will be available for any purpose.
Mechanisms of data availability: The data will be in the public domain.
